# Rationale for osteoporosis screening in men

**DOI:** 10.1007/s00198-024-07337-5

**Published:** 2024-12-13

**Authors:** Radhika R. Narla, Robert A. Adler

**Affiliations:** 1 Division of Endocrinology, Metabolism, and Nutrition, Veterans Affairs Puget Sound Health Care System, Seattle, WA USA; 2 Division of Metabolism, Endocrinology and Nutrition, Department of Medicine, University of Washington School of Medicine, Seattle, WA USA; 3 Endocrinology and Metabolism Section, Richmond Veterans Affairs Medical Center, Central Virginia Veterans Affairs Health Care System, 1201 Broad Rock Boulevard, Richmond, VA 23249 USA; 4 Division of Endocrinology, Metabolism, and Diabetes, Virginia Commonwealth University, Richmond, VA USA

**Keywords:** Bone mineral density, Fracture, Male osteoporosis, Osteoporosis screening, Veterans

## Abstract

**Summary:**

The US Preventive Services Task Force has not recommended osteoporosis screening in men. Department of Veterans Affairs clinicians reviewed the literature on male osteoporosis screening and treatment. They concluded that targeted screening identifies men at risk and osteoporosis drugs reduce fracture risk similarly in men and women.

**Purpose/Introduction:**

The US Preventive Services Task Force (USPSTF) has found insufficient evidence for recommending for or against osteoporosis screening in men. Department of Veterans Affairs osteoporosis experts reviewed the literature on osteoporosis screening and treatment in men.

**Methods:**

Although not done systematically, the literature was reviewed by a panel of 20 Department of Veterans Affairs clinicians with extensive experience with osteoporosis in men. Virtual meetings and multiple email communications resulted in a consensus.

**Results:**

Screening, particularly targeted screening in men, has been found to identify men at risk for fracture. Prior studies have shown osteoporosis drugs have similar effects in men and women. A recent large observational trial demonstrated that hip fracture risk is similarly reduced for both sexes by current medications.

**Conclusion:**

The consensus of the panel was that targeted screening of men for osteoporosis would lead to greater use of osteoporosis medication, lowering fracture risk.

The United States Preventive Services Task Force (USPSTF) has published draft recommendations on screening for osteoporosis. The U.S. Department of Veterans Affairs (VA) Osteoporosis Field Advisory Board (FAB**)** has reviewed the draft statement focusing on guidance regarding case detection and screening for osteoporosis in men. A series of virtual meetings and email communication led to a consensus on whether older men should be screened for osteoporosis. The Osteoporosis FAB is made up of osteoporosis expert clinicians and scientists from Veterans Affairs Healthcare Centers across the country and includes endocrinologists, rheumatologists, geriatricians, neurologists, and health service researchers. Below are comments and the consensus conclusions from the 20 FAB members. Based on the best evidence currently available, the consensus reflects the opinions of members of the FAB and not necessarily the Department of Veterans Affairs.

## Osteoporosis incidence, male osteoporosis epidemiology focusing on veteran population

Amidst an aging population, the importance of osteoporosis prevention and management is becoming increasingly critical. Osteoporosis, a systemic disease marked by low bone mass, deterioration of bone microarchitecture, and skeletal fragility, poses significant health risks. Traditionally, preventive screening for osteoporosis has focused primarily on women, yet we have advocated that men should also be screened for osteoporosis and fracture risk. If screening leads to decreased fracture incidence (and consequences), a proactive approach is essential.

Fragility fractures are the most serious complication of osteoporosis; osteoporosis (defined by bone mineral density) affects approximately 10 million people in the USA, including 2 million men [1]. In 2019, based on emergency room visits, there were just over 200,000 hip fractures in women, but almost 100,000 in men [2]. Case detection, assessing risks, conducting screenings, and initiating treatment discussions are fundamental steps in managing osteoporosis and preventing fractures. These measures are pivotal in reducing the personal and socioeconomic impacts of osteoporotic fracture.

Although 25% of men over the age of 60 will sustain osteoporotic fractures during their lifetime, data suggest that male osteoporosis is underdiagnosed and undertreated. This was observed in a group of > 13,000 adults, especially among those ≥ 70 years old and those at very high risk for osteoporosis [3]. Compared with older women, fewer older men underwent dual‐energy X‐ray absorptiometry (DXA) (12% vs 63%, respectively), and in older men with higher risk (including having already sustained a hip fracture), few men underwent DXA screening (27% versus 36% of women) and 25-OH D measurements (23% versus 28%) and received fewer calcium/vitamin D (40% versus 50%) and bisphosphonate prescriptions (13% versus 24%). The lack of testing and treatment was striking and put these men at significant risk for future bone health complications.

In a recent study [4], hip fracture incidence was found to be increasing among male veterans, and only 6% of those who experienced a fracture had ever undergone DXA. The study suggested that many fractures could have been prevented had a diagnosis of osteoporosis been made earlier. Using the Optum Research Database (which covers persons with commercial insurance or Medicare Advantage plans), a recent study [5] found the fracture rate in men ≥ 65 years old initially declined from 2007 to 2013, but then increased annually with the trend in the fracture rate for the 2014–2017 time period 4.8% higher than the annual trend from 2007 to 2013. This is consistent with the data on male veterans [4].

**Fig. 1:**
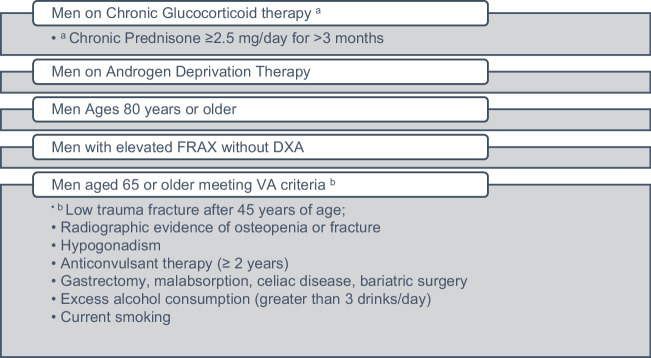
Targeted screening for osteoporosis in men

Another study of older male veterans found that 43% were never treated with an osteoporosis agent, and 90% of those who had received an osteoporosis medication discontinued it [6]. Targeted DXA testing of older men in the largest U.S. integrated health system (the VA) is associated with a lower risk of subsequent fractures in subgroups of men based on clinical characteristics (Fig. 1) [6]. In this observational study using the VA database and Medicare data, the authors found that screening with DXA led to fewer fractures in men on glucocorticoids or androgen deprivation therapy, men at least 80 years of age (with or without other risk factors), men over 65 years old who met a previously published set of criteria for screening veterans [7], or men who were at increased fracture risk based on FRAX calculated without bone density. (FRAX thresholds were ≥ 3% for hip fracture and ≥ 20% for major fracture.) This last-mentioned category is similar to the SCOOP study in women [8]. In this large study done in the UK, only those women at increased risk for fracture based on FRAX without bone density underwent DXA testing. Compared to women not screened at all, this stepwise screening program led to fewer hip fractures. There were more than 12,000 women in the SCOOP study; a similar one in men would likely require 40 to 50 thousand men, which would be prohibitively expensive.

## Osteoporosis-associated mortality

Although delayed by about one decade relative to women, hip fracture incidence in men catches up later in life, especially after age ≥ 75 years. This is particularly concerning because many studies have shown that men have more osteoporosis-related complications and a higher age-adjusted mortality rate after osteoporosis fractures than women. In the prospective cohort from the Dubbo Osteoporosis Epidemiology Study [9] of community-dwelling women and men aged 60 years and older followed for 10 years, the authors found that in women, there were 952 low-trauma fractures followed by 461 deaths (48.4%), and in men, 343 fractures were followed by 197 deaths (57.4%). While the exact etiologies remain unclear, post-fracture infection is one possible explanation for the observed mortality rate.

## New observational study 2024: osteoporosis treatment prevents hip fracture similarly in women and men

In a recent study, Keaveny et al. [10] compared the real-world reduction in risk of hip fracture associated with standard care osteoporosis drug treatment in women vs. men, focusing on patients at high risk of hip fracture (DXA-equivalent hip bone density T-score ≤  − 2.5) and assessing hip fracture outcomes at 2 and 5 years. Using a large integrated health care system electronic health record (EHR), they reviewed all patients ≥ 65 with a hip-containing CT scan for 3 years. The observation started when the patient had the CT scan and extended until the primary outcome of hip fracture, leaving the integrated health system, death, or study end. They reviewed osteoporosis treatment and its impact on hip fracture.

In the final sample from > 11,000 patients, (2413 women and 792 men) using multivariable logistical regression, at 2-year follow-up, 33.9% of the women and 24.0% of the men were treated, primarily with alendronate; 51.3% and 66.3%, respectively, were not-treated; and 721 and 269, respectively, had a first hip fracture since the CT scan. The odds ratio of hip fracture for treated vs. not treated was 0.26 (95% confidence interval 0.21–0.33) for women and 0.21 (0.13–0.34) for men. While the apparent reduction in hip fractures by treatment was surprisingly dramatic, what was important was the ratio of these odds ratios (men/women) of 0.81 (0.47–1.37), indicating no significant sex effect. The authors concluded that the reduction in risk of hip fracture associated with treatment did not differ between the sexes. These results demonstrate that treating osteoporosis in patients at high risk of hip fracture should reduce the risk of hip fracture similarly in men and women. In addition, this study showed the potential utility of trial emulation as a method to derive quality data from large observational studies when randomized controlled trials are not possible, as recently discussed by Blank [11].

In most randomized, controlled (RCT) studies of osteoporosis medications in men, the changes in bone density and bone turnover markers were very similar to the changes observed in similar studies in women. In an RCT with vertebral fracture as the primary outcome, zoledronic acid decreased fractures in men similar to its effect in women [12]. A recent systematic review and meta-analysis [13] concluded that the efficacy of osteoporosis medications was the same in men and women. The mechanisms of action of osteoporosis drugs are the same in males and females.

## Conclusions

One in 3 to 4 hip fractures will occur in men, who also have a higher mortality rate than women after such fractures. Previous inconsistent guidelines have contributed to low screening, testing, and treatment rates for osteoporosis in men, as highlighted by numerous observational studies. The new study by Keaveny et al. [10] demonstrates that treatment reduces the risk of hip fracture equally in both sexes and is consistent with other literature.

Based on this evidence, we strongly advocate that the USPSTF recommend osteoporosis screening in men. Consistent, inclusive guidelines across organizations will bridge screening and treatment gaps, delivering a unified and strengthened message to clinicians and patients about the importance of screening, diagnosing, and treating osteoporosis in men to prevent future fractures and bone health complications. Targeted screening of men at higher risk of fracture because of age, co-morbid conditions, medications associated with osteoporosis, and/or high risk by FRAX should improve osteoporosis management in men. Current practices have led to unnecessary fractures, complications, and deaths.
